# Supplementary material for: Life histories determine divergent population trends for fishes under climate warming
Source: Nat Commun. 2020 Aug 14;11:4088. doi: 10.1038/s41467-020-17937-4 (PMC7428017; doi:10.1038/s41467-020-17937-4)
Supplement: Supplementary file 1 — Supplementary Information [file 41467_2020_17937_MOESM1_ESM.pdf]

Life histories determine divergent population trends for fishes under climate warming

Wang et al.

Supplementary Information

Supplementary Tables (1-9)

Supplementary Figures (1-8)

Supplementary References (1-16)

Supplementary Table 1. Linear mixed-effect model fits for the relationships between a temperature index (mean, max, min, and cv of SST and BT, respectively) and each of life history traits. P-values of fixed- and random-effects, respectively, are evaluated using two-sided *t*- and one-sided χ^2- tests.

a) Mean SST vs. life history traits

Trait	no. pops	β_0, β_1 ($^{\circ -1}$) (Fixed effect)	P-value (fixed effect)	P-values for species and family random effects	R^2 (marginal)	R^2 (conditional)
ln K	1268	-1.26, 0.05	0.001	<0.001, <0.001	0.007	0.83
ln L_∞	1268	3.86, -0.02	<0.001	<0.001, <0.001	0.001	0.95
ln M	1268	-0.62, 0.05	<0.001	<0.001, <0.001	0.006	0.85
ln b	341	1.094, -0.0006	0.90	<0.001, 0.547	$6.70*10^{-5}$	0.46
ln L_{50}	162	3.41, -0.007	0.72	<0.001, <0.001	0.0002	0.93
ln A_{50}	118	1.45, -0.036	0.03	<0.001, <0.001	0.002	0.96
ln A_{max}	194	2.81, -0.02	0.64	<0.001, <0.001	0.004	0.87
b) Maximum SST vs. life history traits						
ln K	1263	-1.25, 0.05	0.002	<0.001, <0.001	0.010	0.84
ln L_∞	1263	3.85, -0.012	<0.001	<0.001, <0.001	0.0007	0.94
ln M	1263	-0.61, 0.006	0.002	<0.001, <0.001	0.0098	0.85
ln b	340	1.09, 0.002	0.72	<0.001, 0.422	0.0008	0.47
ln L_{50}	161	3.41, -0.01	0.63	<0.001, <0.001	0.0003	0.93
ln A_{50}	115	1.34, -0.028	0.38	<0.001, <0.001	0.002	0.96
ln A_{max}	194	2.81, -0.009	0.74	<0.001, <0.001	$7.43*10^{-5}$	0.87

c) Minimum SST vs. life history traits

Trait	no. pops	β_0, β_1 ($^{\circ -1}$) (Fixed effect)	P-value (fixed effect)	P-values for species and family random effects	R^2 (marginal)	R^2 (conditional)
ln K	1263	-1.26, 0.033	0.002	<0.001, <0.001	0.005	0.82
ln L_∞	1263	3.86, -0.022	0.0002	<0.001, <0.001	0.003	0.95
ln M	1263	-0.608, 0.04	0.001	<0.001, <0.001	0.005	0.84
ln b	340	1.09, -0.002	0.59	<0.001, 0.805	0.001	0.45
ln L_{50}	161	3.41, -0.013	0.45	<0.001, <0.001	0.0008	0.92
ln A_{50}	115	1.36, -0.031	0.0037	<0.001, <0.001	0.002	0.95
ln A_{max}	194	2.81, -0.03	0.16	<0.001, <0.001	0.001	0.88

d) cv SST vs. life history traits

ln K	1263	-1.26, -0.83	0.05	<0.001, <0.001	0.0006	0.81
ln L_∞	1263	3.85, 0.45	0.02	<0.001, <0.001	0.0002	0.94
ln M	1263	-0.607, -0.971	0.03	<0.001, <0.001	0.0006	0.83
ln b	340	1.094, -0.068	0.66	<0.001, 0.623	0.0003	0.41
ln L_{50}	161	3.42, 2.22	0.003	<0.001, 0.001	0.005	0.91
ln A_{50}	115	1.36, -0.23	0.76	<0.001, <0.001	$4.19*10^{-5}$	0.95
ln A_{max}	194	2.81, 4.003	0.25	<0.001, <0.001	0.0009	0.88

e) Mean BT vs. life history traits.

Trait	no. pops	β_0, β_1 ($^{\circ -1}$) (Fixed effect)	P-value (fixed effect)	P-values for species and family random effects	R^2 (marginal)	R^2 (conditional)
ln K	1190	-1.28, 0.03	0.03	<0.001, <0.001	0.005	0.84
ln L_∞	1190	3.88, -0.006	0.03	<0.001, <0.001	0.0002	0.94
ln M	1190	-0.63, 0.04	0.02	<0.001, <0.001	0.005	0.85
ln b	306	1.093, $1.16*10^{-3}$	0.56	<0.001, 0.595	0.0007	0.40
ln L_{50}	157	3.45, -0.02	0.06	<0.001, 0.002	0.002	0.90
ln A_{50}	117	1.45, -0.06	0.004	<0.001, <0.001	0.003	0.96
ln A_{max}	165	2.81, -0.04	0.03	<0.001, <0.001	0.003	0.88

f) Maximum BT vs. life history traits

ln K	1190	-1.28, 0.03	0.01	<0.001, <0.001	0.065	0.84
ln L_∞	1190	3.88, -0.007	0.002	<0.001, <0.001	0.0004	0.94
ln M	1190	-0.63, 0.04	0.008	<0.001, <0.001	0.006	0.85
ln b	306	1.093, $9.55*10^{-5}$	0.97	<0.001, 0.596	$3.72*10^{-6}$	0.40
ln L_{50}	157	3.45, -0.009	0.72	0.001, 0.002	0.0003	0.92
ln A_{50}	117	1.45, -0.02	0.39	<0.001, <0.001	0.0003	0.96
ln A_{max}	165	2.80, -0.07	0.23	<0.001, <0.001	0.007	0.91

g) Minimum BT vs. life history traits

Trait	no. pops	β0, β1 (°-1) (Fixed effect)	P-value (fixed effect)	P-values for species and family random effects	R^2 (marginal)	R^2 (conditional)
ln K	1190	-1.28, 0.03	0.05	<0.001, <0.001	0.006	0.84
ln L_∞	1190	3.88, -0.016	0.0045	<0.001, <0.001	0.002	0.95
ln M	1190	-0.63, 0.04	0.03	<0.001, <0.001	0.006	0.85
ln b	305	1.093, $9.54*10^{-4}$	0.55	<0.001, 0.595	0.0007	0.40
ln L_{50}	156	3.45, -0.03	0.17	<0.001, 0.022	0.006	0.93
ln A_{50}	114	1.36, -0.07	0.002	<0.001, <0.001	0.042	0.96
ln A_{max}	165	2.81, -0.03	0.05	<0.001, <0.001	0.003	0.88

h) cv BT vs. life history traits

ln K	1185	-1.28, 0.177	0.66	<0.001, <0.001	0.0003	0.82
ln L_∞	1185	3.88, -0.07	0.23	<0.001, <0.001	$7.09*10^{-5}$	0.94
ln M	1185	-0.64, 0.071	0.87	<0.001, <0.001	$3.79*10^{-5}$	0.84
ln b	305	1.09, -0.075	0.09	<0.001, 0.607	0.006	0.41
ln L_{50}	156	3.45, 0.177	0.51	<0.001, 0.002	0.0003	0.90
ln A_{50}	114	1.36, 0.52	0.17	<0.001, <0.001	0.0008	0.95
ln A_{max}	165	2.81, 1.09	0.26	<0.001, <0.001	0.00096	0.88

Supplementary Table 2. Linear mixed-effect model fits of the effect of mean SST on the scores of the first principal component (PC1) derived from PCA analyses with 3 sets of life history traits. For the PC1 scores derived from PCA of ln *K* and ln L_∞ with the largest sample size (in bold), we found a significant negative temperature effect on the PC1, indicative of a positive temperature effect on ln *K* while a negative effect on ln L_∞. These results are consistent with the analysis in the panel a) of Table S1. P-values of fixed- and random-effects, respectively, are evaluated using two-sided *t*- and one-sided χ^2- tests.

Trait	no. pops	β_0, β_1 ($^{\circ -1}$) (Fixed effect)	P-value (fixed effect)	P-values for species and family random effects	PC1 Loadings
PC1 (ln *K*, ln L_∞, ln *M*, and ln *b*)	333	-0.154, -0.06	0.09	<0.001, 0.004	(-0.62, 0.45, -0.64, 0.05) for (ln *K*, ln L_∞, ln *M*, and ln *b*)
PC1 (ln A_{50} and ln L_{50})	85	-0.08, -0.04	0.41	<0.001, 0.009	(0.71, 0.71) for (ln A_{50} and ln L_{50})
**PC1 (ln *K* and ln L_∞)**	**1277**	**0.129, -0.05**	**<0.001**	<0.001, <0.001	**(-0.71, 0.71) for (ln *K* and ln L_∞)**

Supplementary Table 3. Mean ($\bar{x}$) and 95 % confidence intervals (CIs) of covariance coefficients between temperature and each of the six life history traits (on log-scale) for 302 species in our database using the multivariate modeling approach by Thorson et al. (2017). K: Brody growth coefficient L_∞: asymptotic length, M: natural mortality, A_{max}: maximum age, A_{mat}: age at maturity, and L_{mat}: length at maturity.

	$\bar{x}$	95 % CI
ln K	0.204	(0.172, 0.236)
ln L_∞	-0.046	(-0.059, -0.033)
ln M	0.261	(0.223, 0.299)
ln A_{max}	-0.169	(-0.197, -0.141)
ln A_{mat}	-0.158	(-0.186, -0.130)
ln L_{mat}	-0.100	(-0.116, -0.084)

Supplementary Table 4. Generalized additive mixed-effect model fits for evaluating the relationships between mean SST and each of life history traits (with species as a random-effect variable). P-values of fixed-effects are evaluated using two-sided t-tests, and the approximate P-values of smooth terms are evaluated using one-sided F-tests.

Trait	no. pops	β_0 (Fixed effect)	P-value (β_0)	s(SST)	P-value (s(SST))
ln K	1268	-1.18	<0.001	9.46	<0.001
ln L_∞	1268	3.71	<0.001	8.52	<0.001
ln M	1268	-0.5	<0.001	9.72	<0.001
ln b	341	1.09	<0.001	0.01	0.91
ln L_{50}	162	3.44	<0.001	3.02	0.08
ln A_{50}	118	1.36	<0.001	4.73	0.03
ln A_{max}	194	1.03	<0.001	0.2	0.99

Supplementary Table 5. Definition of six fish groups based on *FishBase*.

Pelagic fishes	Fishes that live and feed in the open sea, are associated with the surface or middle depths of a body of water, freely swim in the seas, oceans or open waters, and are not in association with the bottom. Pelagic zone generally refers to 0-200 m of depth.
Benthopelagic fishes	Fishes that live and feed near the bottom as well as in midwaters or near the surface. Some may feed or swim on or over the floor of the sea. The depth range includes 100 m off the bottom at all depths below the edge of the continental shelf.
Reef fishes	Fishes that live and feed on or near coral reefs.
Elasmobranch fishes	Chondrichthyes, including sharks and rays.
Demersal fishes	Fish that live on or near bottom of the sea and they feed on benthic organisms.
Bathydemersal fishes	Fishes that live and feed on or near bottom of the sea below 200 m.

Supplementary Table 6. Linear mixed-effect model fits for the relationships between mean SST and each life history trait by six groups of fishes (a-g). Also, model fits for the effect of mean BT on each trait are provided (h-n). P-values of fixed-effects are evaluated using two-sided *t*-tests.

a) Mean SST vs. ln *K*

Fish group	No. pops	β_0	β_1 (°$^{-1}$)	Se (β_0)	Se (β_1)	P-value (*: ≤0.05)
Bathydemersal	99	-2.28	0.01	0.176	0.012	0.34
Demersal	240	-1.05	0.07	0.141	0.031	0.06
Elasmobranch	96	-2.29	0.06	0.118	0.024	0.02*
Benthopelagic	117	-1.42	0.05	0.238	0.020	0.03*
Pelagic	176	-0.91	0.03	0.154	0.027	0.24
Reef	460	-0.76	0.08	0.155	0.017	<0.001*

b) Mean SST vs. ln *M*

Bathydemersal	99	-1.66	0.02	0.178	0.013	0.22
Demersal	240	-0.34	0.08	0.163	0.034	0.06
Elasmobranch	96	-1.86	0.06	0.127	0.026	0.02*
Benthopelagic	117	-0.77	0.05	0.239	0.022	0.02*
Pelagic	176	-0.29	0.03	0.194	0.031	0.32
Reef	460	-0.014	0.09	0.192	0.019	<0.001*

c) Mean SST vs. ln L_∞

Bathydemersal	99	3.80	-0.02	0.210	0.004	<0.001*
Demersal	240	3.51	-0.02	0.123	0.006	0.01*
Elasmobranch	96	4.77	-0.01	0.165	0.012	0.41
Benthopelagic	117	3.74	-0.03	0.158	0.009	0.003
Pelagic	176	3.99	-0.01	0.203	0.014	0.58
Reef	460	3.52	-0.03	0.116	0.010	0.006*

d) Mean SST vs. ln b

Bathydemersal	4	na	na	na	na	na
Demersal	63	1.10	-0.003	0.025	0.008	0.67
Elasmobranch	14	1.14	-0.008	0.018	0.006	0.27
Benthopelagic	47	1.11	0.02	0.017	0.014	0.29
Pelagic	43	1.06	0.002	0.028	0.010	0.89
Reef	133	1.09	-0.01	0.016	0.005	0.05*

e) Mean SST vs. ln L_{50}

Bathydemersal	23	3.53	$7.73*10^{-4}$	0.153	$1.19*10^{-2}$	0.95
Demersal	18	3.61	-0.03	0.150	0.090	0.76
Elasmobranch	19	4.37	-0.06	0.247	0.020	0.04*
Benthopelagic	14	3.48	-0.09	0.179	0.300	0.77
Pelagic	22	3.16	0.04	0.169	0.158	0.79
Reef	61	3.16	0.03	0.109	0.026	0.32

f) Mean SST vs. ln A_{50}

Bathydemersal	22	2.59	-0.010	0.299	0.030	0.68
Demersal	27	1.64	-0.030	0.118	0.040	0.49
Elasmobranch	11	2.92	0.007	0.188	0.123	0.96
Benthopelagic	5	na	na	na	na	na
Pelagic	4	na	na	na	na	na
Reef	48	0.95	-0.103	0.088	0.096	0.29

g) Mean SST vs. ln A_{max}

Bathydemersal	7	4.09	0.19	0.206	0.130	0.21
Demersal	9	1.98	-0.11	0.269	0.220	0.64
Elasmobranch	na	na	na	na	na	na
Benthopelagic	3	na	na	na	na	na
Pelagic	na	na	na	na	na	na
Reef	146	2.85	-0.06	0.171	0.028	0.04*

h) Mean BT vs. ln *K*

Fish group	No. pops	β_0	β_1 (°⁻¹)	Se (β_0)	Se (β_1)	P-value (*: ≤0.05)
Bathydemersal	99	-2.28	-0.08	0.172	0.072	0.30
Demersal	240	-1.05	0.05	0.140	0.042	0.27
Elasmobranch	96	-2.31	0.007	0.102	0.023	0.78
Benthopelagic	117	-1.42	0.02	0.237	0.011	0.17
Pelagic	176	-0.83	0.03	0.127	0.020	0.22
Reef	460	-0.76	0.07	0.156	0.027	0.03*

i) Mean BT vs. ln *M*

Bathydemersal	99	-1.66	-0.08	0.178	0.074	0.32
Demersal	240	-0.34	0.06	0.162	0.045	0.25
Elasmobranch	96	-1.88	0.007	0.112	0.025	0.77
Benthopelagic	117	-0.77	0.02	0.239	0.012	0.15
Pelagic	176	-0.19	0.02	0.157	0.022	0.28
Reef	460	-0.01	0.08	0.192	0.029	0.02*

j) Mean BT vs. ln L_∞

Bathydemersal	99	3.74	0.006	0.170	0.022	0.79
Demersal	240	3.52	-0.003	0.123	0.006	0.57
Elasmobranch	96	4.77	-0.005	0.165	0.011	0.68
Benthopelagic	117	3.74	-0.002	0.158	0.005	0.69
Pelagic	176	4.02	-0.004	0.210	0.007	0.57
Reef	460	3.52	-0.016	0.116	0.008	0.03*

k) Mean BT vs. ln b

Bathydemersal	4	na	na	na	na	na
Demersal	63	1.11	0.002	0.024	0.008	0.82
Elasmobranch	14	1.14	-0.005	0.018	0.004	0.23
Benthopelagic	47	1.11	0.008	0.017	0.004	0.09
Pelagic	43	1.06	0.006	0.028	0.004	0.20
Reef	133	1.09	-0.008	0.009	0.010	0.45

l) Mean BT vs. ln L_{50}

Bathydemersal	23	3.53	0.03	0.153	0.020	0.16
Demersal	18	3.74	-0.04	0.239	0.067	0.54
Elasmobranch	19	4.36	-0.03	0.246	0.029	0.33
Benthopelagic	14	3.48	-0.01	0.180	0.067	0.90
Pelagic	22	3.35	0.03	0.342	0.033	0.35
Reef	61	3.15	-0.07	0.088	0.022	0.005*

m) Mean BT vs. ln A_{50}

Bathydemersal	22	2.59	-0.04	0.299	0.068	0.60
Demersal	27	1.55	-0.07	0.062	0.077	0.36
Elasmobranch	11	2.92	0.01	0.188	0.150	0.96
Benthopelagic	5	na	na	na	na	na
Pelagic	4	na	na	na	na	na
Reef	48	0.91	-0.08	0.113	0.022	0.001*

n) Mean BT vs. ln A_{max}

Bathydemersal	7	4.09	-0.11	0.232	0.139	0.47
Demersal	9	1.98	-0.19	0.269	0.392	0.64
Elasmobranch	na	na	na	na	na	na
Benthopelagic	3	na	na	na	na	na
Pelagic	na	na	na	na	na	na
Reef	146	2.82	-0.12	0.170	0.052	0.04*

Supplementary Table 7. The equations for conversions between different length metrics among studies. Notations of each type of length metrics: TL: total length, PL: pre-anal length (horizontal distance from the tip of upper jaw to anus), AL: pre-anal length (horizontal distance from the tip of lower jaw to anus), FL: fork length, and SL: standard length.

Species	Equation	Length range (cm)	Reference
Trichiurus lepturus	TL = 4.85 + 2.56 * PL	-	First author's unpublished data
Trichiurus lepturus	PL = -0.05 + 0.978 * AL	-	First author's unpublished data (based on *T. japonicus*)
Decapterus russelli	FL = 0.92 * TL	-	(*1*)
Decapterus macrosoma	TL = -0.22 + 1.1 * FL	-	(*1*)
Psenopsis anomala	SL = 0.702 * TL	-	(*1*)
Psenopsis anomala	FL = 0.794 * TL	-	(*1*)
Muraenesox cinereus	PL = 0.388 * TL	-	(*1*)
Pampus argenteus	TL = 1.265 * FL	-	(*1*)
Pennahia argentata	TL = 1.176 * SL	-	(*1*)
Priacanthus macracanthus	TL = 1 * FL	-	(*1*)
Priacanthus macracanthus	TL = 1.1389 * SL	-	(*1*)
Ctenochaetus striatus	SL = 0.739 * TL	12.7-16.4	(*1*)
Ctenochaetus striatus	SL = 0.767 * TL	20.5-22.5	(*1*)
Ctenochaetus striatus	TL = 1.136 * FL	-	(*1*)
Ctenochaetus striatus	TL = 1.309 * SL	-	(*1*)
Encrasicholina heteroloba	FL = 0.911 * TL	-	(*1*)
Engraulis mordax	FL = 0.905 * TL	-	(*2*)
Engraulis mordax	SL = 0.942 * FL	-	(*2*)
Engraulis mordax	SL = 0.853 * TL	-	(*2*)
Cetengraulis mystucetus	TL = 1.25 * SL	-	(*3*)
Dentex tumifrons	TL = 1.088 * FL	-	(*1*)
Acanthurus lineatus	SL = -0.38 + 0.86 * FL	-	(*4*)
Lutjanus sebae	FL = 11.823 + 1.1521 * SL	-	(*5*)
Lutjanus malabaricus	SL = -1.106 + 0.829 * FL	-	(*5*)
Chlorurus sordidus	SL = -0.213 + 0.878 * TL	-	(*1*)
Chlorurus sordidus	FL = 1 * TL	-	(*1*)
Scarus frenatus	FL = 0.8803 * SL	-	(*1*)
Scarus frenatus	FL = 1 * TL	-	(*1*)
Lethrinus nebulosus	FL = -0.84 + 1.12 * SL	-	(*6*)

Species	Equation	Length range (cm)	Reference
Chlorurus microrhinos	TL = 1.188 * FL	-	(*1*)
Chlorurus microrhinos	TL = 1.343 * SL	-	(*1*)
Lutjanus fulviflamma	TL = 1.033 * FL	-	(*1*)
Lutjanus fulviflamma	TL = 1.164 * SL	-	(*1*)
Hipposcarus longiceps	FL = 1.146 * SL	-	(*1*)
Hipposcarus longiceps	FL = 1.146 * SL	-	(*1*)
Scarus psittacus	SL = 0.833 * TL	28-36	(*1*)
Scarus psittacus	FL= 1 * TL	28-36	(*1*)
Siganus fuscescens	TL = 1.189 * SL	-	(*1*)
Acanthurus nigrofuscus	FL = 1.253 * SL	-	(*1*)
Naso lituratus	FL = 1.134 * SL	-	(*1*)
Naso unicornis	FL = 1.125 * SL	-	(*1*)
Cetoscarus bicolor	FL = 1.091 * SL	-	(*1*)
Scarus altipinnis	FL = 1.1 * SL	-	(*1*)
Scarus niger	FL = -2.443 + 1.336 * SL	-	(*7*)
Scarus rivulatus	FL = 1.1494 * SL	-	(*1*)
Scarus schlegeli	FL = 1.104 * SL	-	(*1*)
Scarus ghobban	FL = 0.915 * TL	-	(*1*)
Hipposcarus harid	TL = 1.168 * SL	-	(*1*)
Hipposcarus harid	TL = 1.063 * FL	-	(*1*)
Siganus sutor	FL = 0.975 * TL	-	(*1*)
Lethrinus lentjan	FL = 0.902 * TL	-	(*1*)
Lethrinus mahsena	FL = 0.932 * TL	-	(*1*)
Aphareus rutilans	FL = 1.052 * SL	-	(*1*)
Rhabdosargus sarba	FL = 0.95 * TL	-	(*1*)
Lethrinus nebulosus	FL = 0.312 + 0.89 * TL	-	(*6*)
Bolbometopon muricatum	TL = 1 * FL	-	(*1*)
Bolbometopon muricatum	TL = 1.178 * SL	-	(*1*)

Supplementary Table 8. Methods of estimation of natural mortality for the fish populations in this study. Trait abbreviations: M = natural mortality; L_∞ and K = asymptotic length and growth coefficient, respectively, in von Bertalanffy growth equation; T_{max} = maximal age; T_{mat} = age at sexual maturation; Z = total mortality.

Method	Equation	Reference
Pauly	$\log M = -0.0066 - 0.279 \log L_\infty + 0.6543 \log K + 0.4634 \log \text{Temperature}$	(*8*)
Cushing	$M = (\ln 100)/T_{max}$	(*9*)
Catch curve*	$\ln (N_t) = \ln (N_0) - Zt$	(*10*)
Hoenig	$\ln Z = 1.46 - 1.01 \ln T_{max}$	(*12*)
Beverton and Holt	Empirical relationship between *M* and *K* (no equations).	(*11*)
Ralston	$M = 0.0189 + 2.06\, K$	(*13*)
Rickhter and Efanov	$M = [0.72\ (1.52/T_{mat})\ ] - 0.16$	(*14*)
Mathews and Samuel	$M = 0.5\, Z$	(*15*)
Alverson and Carney	$0.38^*\ T_{max} = (1/K) \ln ((M+3K)/M)$	(16)

* Catch curve method is applied for unexploited species.

Supplementary Table 9. Linear mixed-effect model fits for the relationships between mean SST and each of life history traits based on data excluding the 34 species with relatively large variation in *K* and L_∞ among populations. These results are consistent with Table S1 (panel a). P-values of fixed- and random-effects, respectively, are evaluated using two-sided *t*- and one-sided χ^2- tests.

Traits	no. pops	β_0, β_1 (°$^{-1}$) (Fixed effect)	P-value (fixed effect)	P-values for species and family random effects
ln K	879	-1.24, 0.06	0.004	<0.001, <0.001
ln L_∞	879	3.79, -0.02	<0.001	<0.001, <0.001
ln M	879	-0.58, 0.06	0.003	<0.001, <0.001
ln b	235	1.09, -0.006	0.10	<0.001, 0.987
ln L_{50}	115	3.28, -0.03	0.04	<0.001, 0.002
ln A_{50}	75	1.54, -0.04	0.004	<0.001, <0.001
ln A_{max}	156	2.79, -0.02	0.64	0.001, <0.001

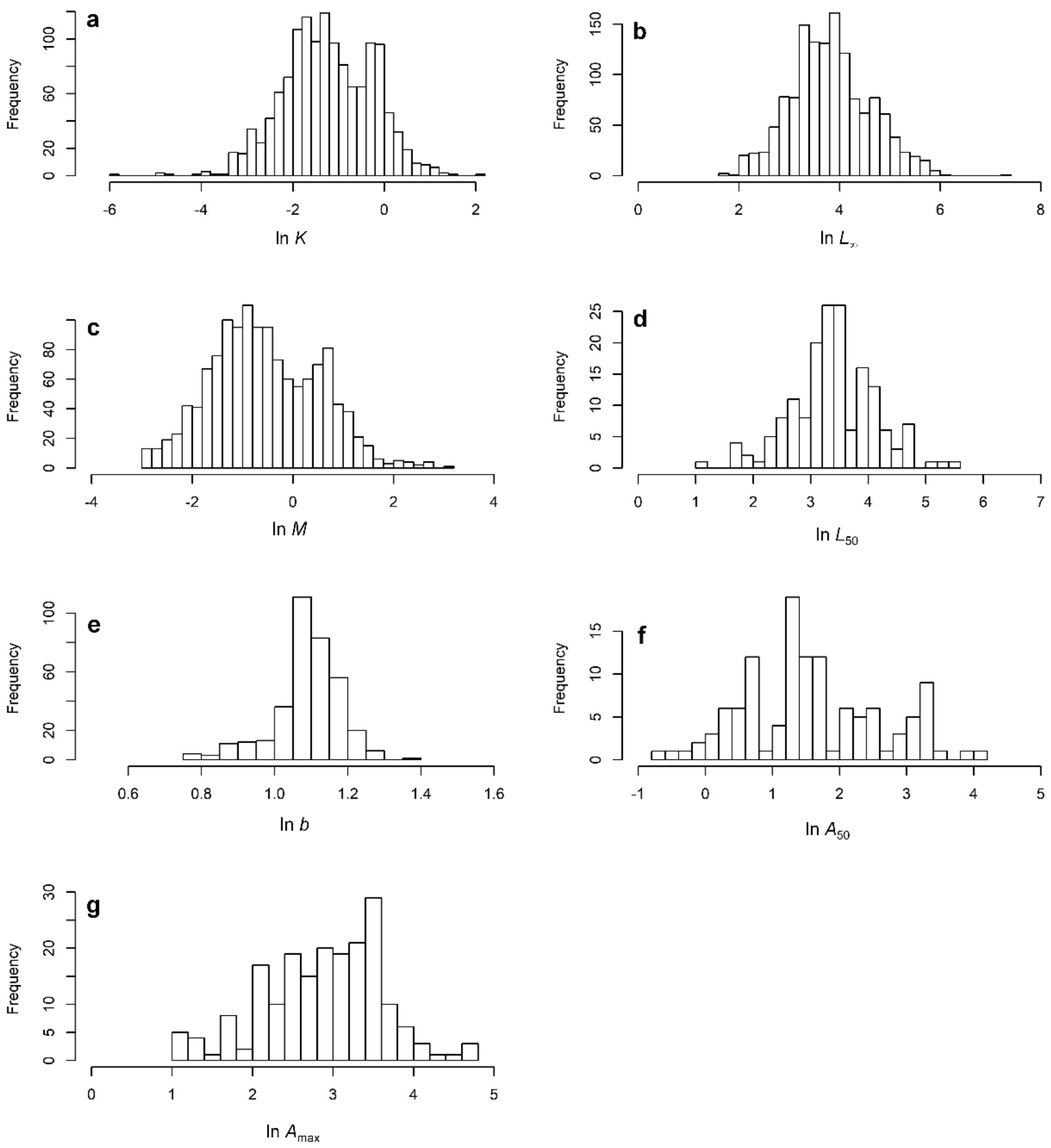

Supplementary Figure 1. Histograms of population life history parameters in our database. The x-axes are the parameters on the natural-log scales. Number of populations for each trait: n = 1,341 ($\ln K$), 1,343 ($\ln L_\infty$), 1,330 ($\ln M$), 166 ($\ln L_{50}$), 357 ($\ln b$), 119 ($\ln A_{50}$), and 194 ($\ln A_{max}$).

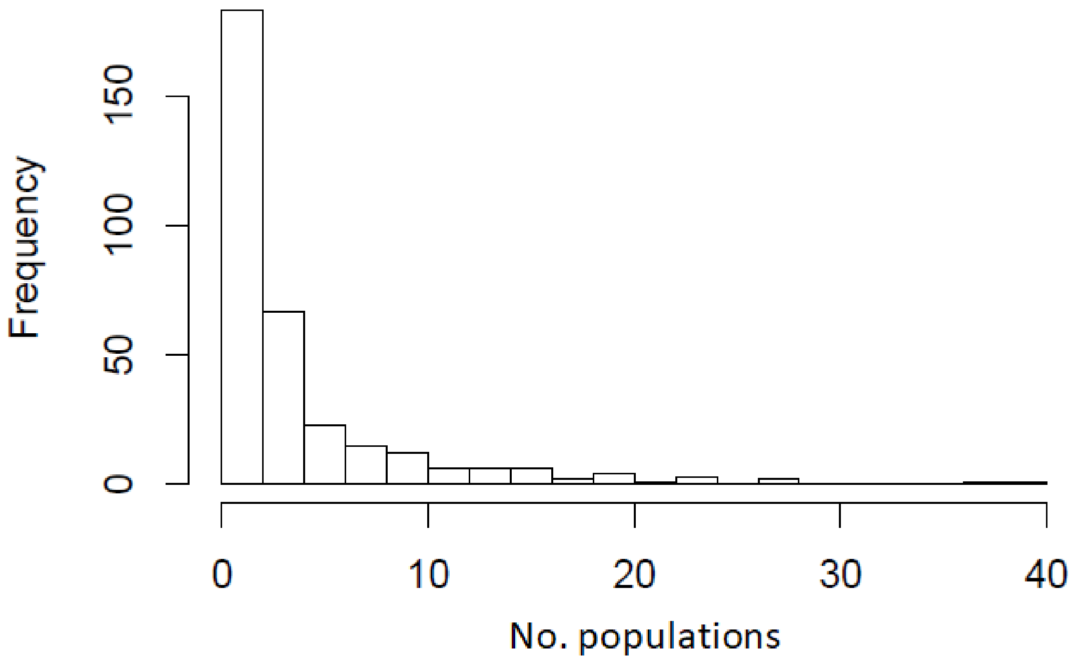

Supplementary Figure 2. Distribution of number of populations per species in our database. Total n = 1,402 populations.

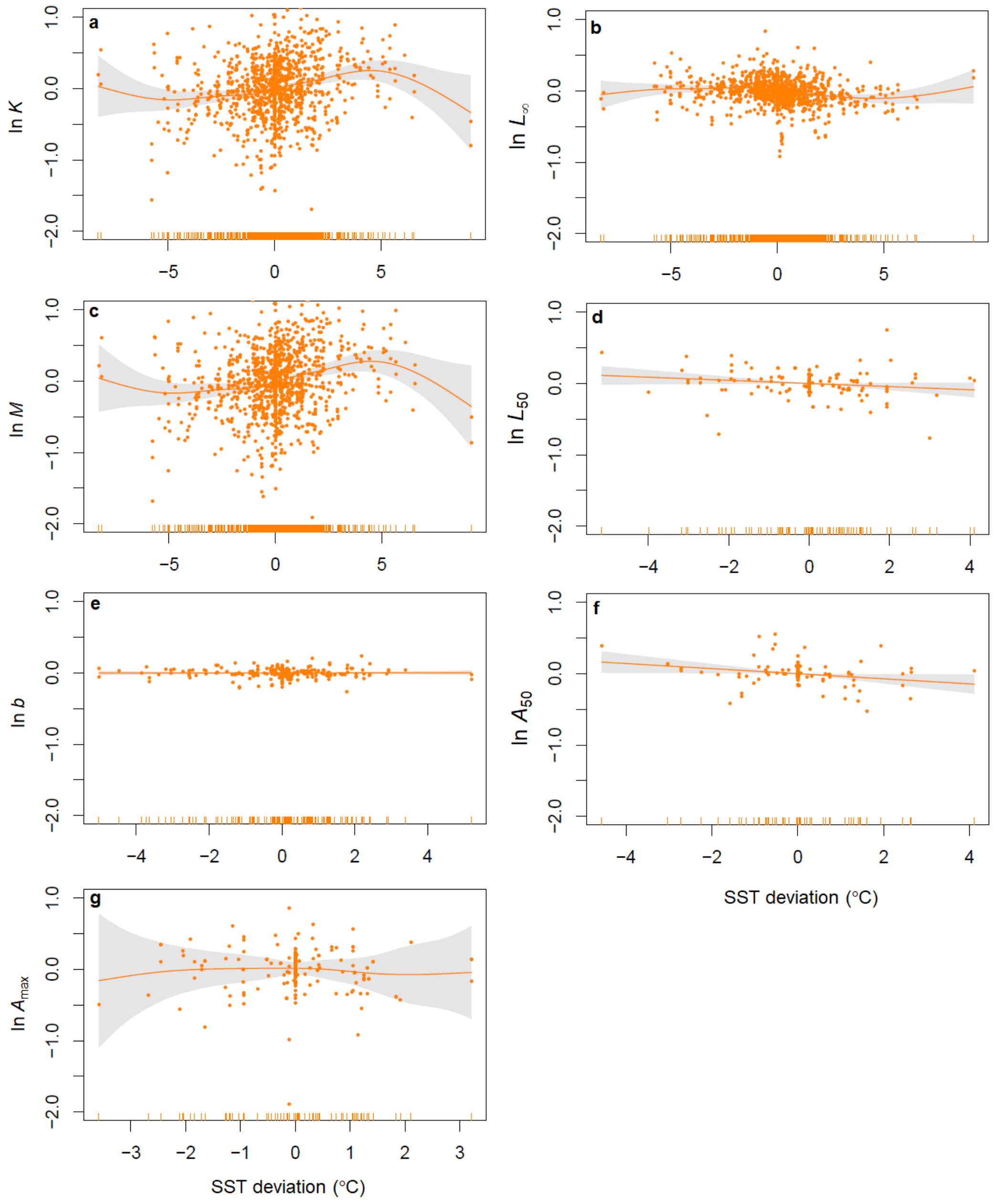

Supplementary Figure 3. Generalized additive mixed-effect model fits to evaluate nonlinear temperature effects on each of the life history trait. Temperature effects are generally linear within ±5 °C from the mean temperature.

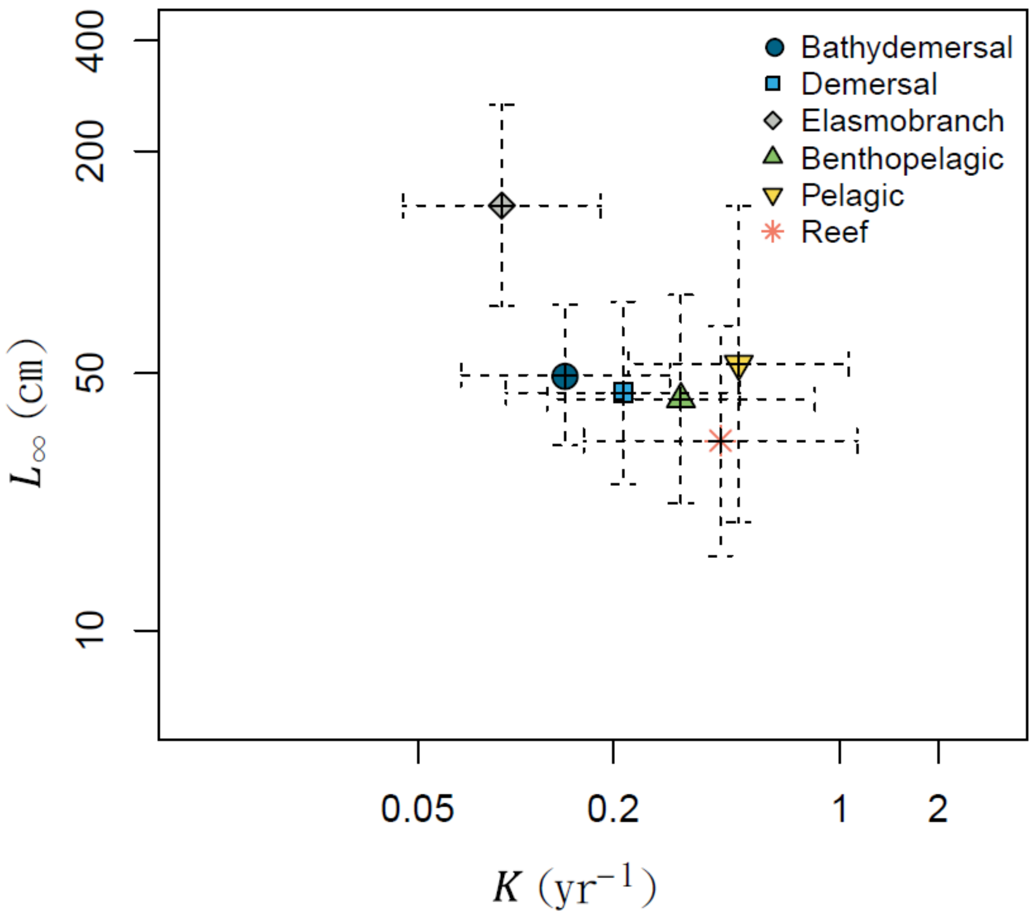

Supplementary Figure 4. A bi-variate plot showing mean values +/- SD (shown as error bars) of population traits K and L_∞ for each of the six groups of fishes in our database. Number of populations for each group: n = 101 (bathydemersal), 280 (demersal), 100 (elasmobranch), 141 (benthopelagic), 206 (pelagic), and 526 populations (reef).

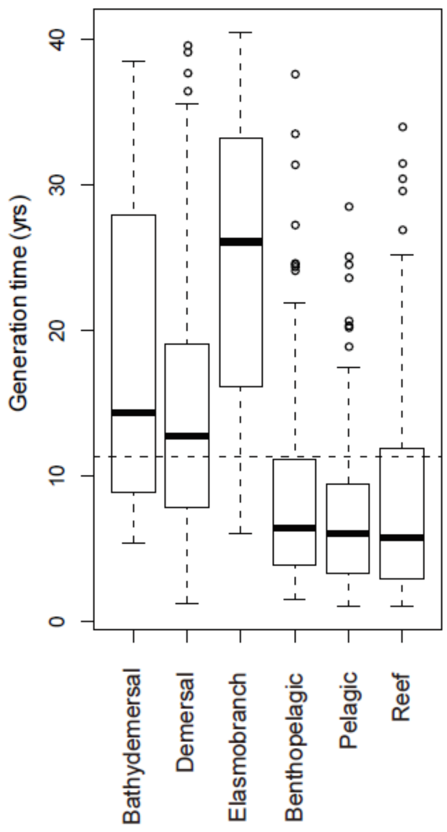

Supplementary Figure 5. Boxplots of estimated generation time (yrs) derived from population life table models for each habitat-related fish group. The horizontal dashed line denotes the mean generation time = 11.4 yrs for all populations. The lower bounds, centers, and upper bounds of the boxes, respectively, correspond to the 25th, 50th, and 75th percentile of data values within each group. The whiskers extend to the most extreme data point no more than 1.5 times the interquartile range of data within each group. Number of populations for each group: n = 97 (bathydemersal), 254 (demersal), 97 (elasmobranch), 126 (benthopelagic), 178 (pelagic), and 474 populations (reef).

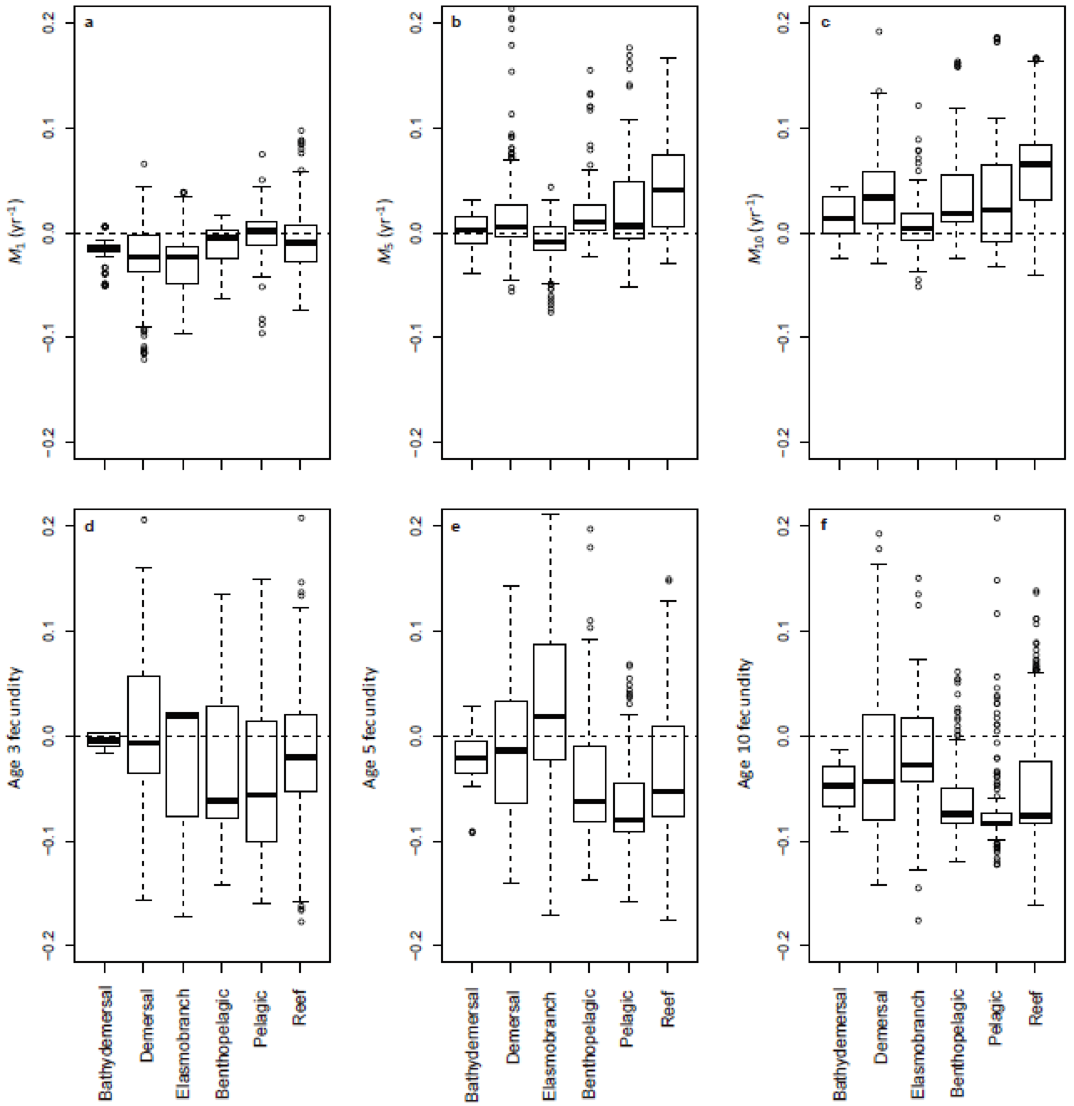

Supplementary Figure 6. Model-derived log (base 2) ratios of rising 1°C vs. the baseline mean temperature for the age-specific natural mortality (panel a-c) and fecundity (d-f) derived from population life table models for each habitat-related fish group. We selected to plot only 3 age classes to facilitate visualization of data (in total, 50 age classes). For the fish groups with slower life histories (bathydemersal, demersal, and elasmobranch fishes), rising temperature leads to lower natural mortality and no or positive changes in fecundity at younger ages, while these patterns reversed at older ages. On the other hand, rising temperature leads to no changes or increased natural mortality and decreased fecundity at all ages for the fish groups with fast life histories (benthopelagic, pelagic, and reef fishes). The lower bounds, centers,

and upper bounds of the boxes, respectively, correspond to the 25th, 50th, and 75th percentile of data values within each group. The whiskers extend to the most extreme data point no more than 1.5 times the interquartile range of data within each group. Number of populations for each group: n = 97 (bathydemersal), 254 (demersal), 97 (elasmobranch), 126 (benthopelagic), 178 (pelagic), and 474 populations (reef).

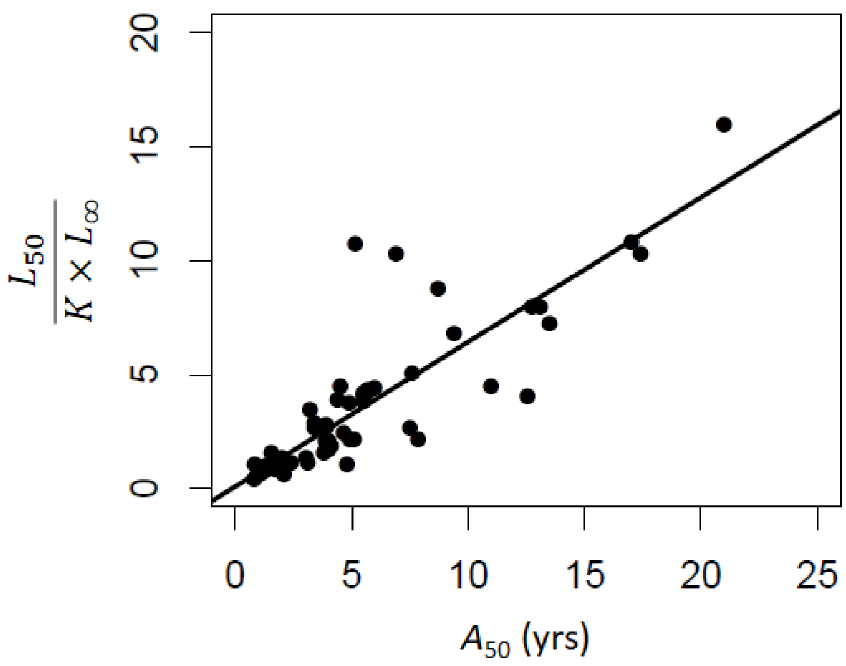

Supplementary Figure 7. A linear regression fit for the empirical relationship between population A_{50} and the $\frac{L_{50}}{K \times L_{\infty}}$ based on 70 populations in our database.

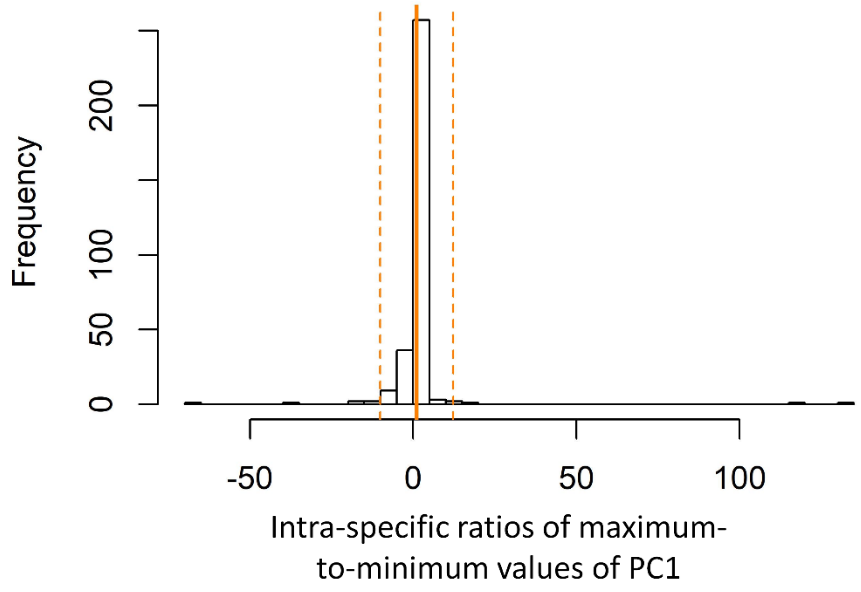

Supplementary Figure 8. Distribution of the species ratios of maximum-to-minimum values of the scores of the first principle component (PC1) derived from the PCA of a combination of 2 traits with sufficient population data: ln K and ln L_∞. Solid and dashed vertical lines indicate mean (1.06) and mean ± 1 standard deviation (11.17) of the PC1 scores. Thirty-four species are found with |ratios| ≥3, while 285 species with |ratios| <3.

Supplementary References

1. Froese, R, & Pauly D., Eds. FishBase. World Wide Web electronic publication. www.fishbase.org, version (2019).

2. Whitehead, P. J. P. Clupeoid Fishes of the World (Suborder Clupeoidei): An Annotated and Illustrated Catalogue of the Herrings, Sardines, Pilchards, Sprats, Shads, Anchovies, and Wolfherrings. Part 1 –Clupeididae. *FAO Fish Synop.*, Rome (1985).

3. Bayliff, W. H. Growth, mortality, and exploitation of the Engraulidae, with species reference to the anchoveta, *Cetengraulis mysticetus*, and the colorado, *Anchoa naso*, in the eastern Pacific Ocean. *IATTC Bull.* **12**(5), 365-432 (1967).

4. Craig, P. C., Choat, J. H., Axe, L. M., & Saucerman, S. Population biology and

harvest of the coral reef surgeonfish *Acanthurus lineatus* in American Samoa. *Fish. Bull.* **95**, 680-693 (1997).

5. Newman, S. J. & Dunk, I. J. Growth, age validation, mortality, and other population characteristics of the red emperor snapper, *Lutjanus sebae* (Cuvier, 1828), off the Kimberley coast of north-western Australia. *Estuar. Coast. Shelf Sci.* **55**, 67-80 (2002).

6. Loubens, G. Biologie de quelques espe'ces de Poissons du lagon Ne'o-Cale'donien. III. Croissance. *Cah Indo-Pac* **2**, 101-153 (1980).

7. Gumanao, G. S., Saceda-Cardoza, M. M., Mueller, B., & Bos, A. R. Length-weight and length-length relationships of 139 Indo-Pacific fish species (Teleostei) from the Davao Gulf, Philippines. *J. Appl. Ichthyol.* doi: 10.1111/jai.12993 (2016).

8. Pauly, D. On the interrelationships between natural mortality, growth parameters, and mean environmental temperature in 175 fish stocks. *J. Cons. Int. Explor. Mer.* **39**, 175-192 (1980).

9. Cushing, D. H. Fisheries biology: a study in population dynamics. (Univ. Wisconsin Press, Madison, 1968).

10 Beverton, R. J. H. & Holt, S. J. On the dynamics of exploited fish populations. *Fish. Investigat.* **19**, 1-533 (1957).

11 Beverton, R. J. H. & Holt, S. J. A review of the lifespans and mortality rates of fish in nature, and their relation to growth and other physiological characteristics. In: *Ciba Foundation Colloquia on ageing V, The lifespan of animals*, G. E. W. Wolstenholme, M. O'Connor, Eds. 142-177 (1959).

12 Hoenig, J. M. Empirical use of longevity data to estimate mortality rates. *Fish. Bull.* **82**, 898-903 (1983).

13. Ralston, S. Mortality rates of snappers and groupers. In: *Tropical Snappers and*

Groupers: Biology and Fisheries Management, J. J. Polovina, S. Ralston, Eds., 375-404 (Westview Press, Boulder, 1987).

14. Rickhter, V. A. & Efanov, V. N. On one of the approaches to estimation of natural mortality of fish populations. *ICNAF Res. Doc.* 76/VI/8, 1:12 (1976).

15. Mathews, C. P. & Samuel, M. Stock assessment and management of newaiby, hamoor and hamra in Kuwait. In: *Final Report- Proceedings of the 1984 Shrimp and Fin Fisheries Management Workshop*, C. P. Mathews, Ed., 67-115 (Kuwait Institute for Scientific Research, Kuwait, MB-51, 1985).

16. Alverson, D. L. & Carney, M. J. A graphic review of the growth and decay of population cohorts. *J. Cons. Int. Explor. Mer* **36**,133-143 (1975).
